# Concentrations, Compositions and Human Exposure Risks to Organophosphate Esters in Indoor Air from Various Microenvironments in Guangzhou, China

**DOI:** 10.3390/toxics13070531

**Published:** 2025-06-25

**Authors:** Yunmei Cai, Maoyuan Xu, Minghui Ouyang, Yusheng Wu, Ruijie Wang, Kewen Zheng, Guofa Ren

**Affiliations:** 1 School of Environmental Monitoring, Guangdong Polytechnic of Environmental Protection Engineering, Foshan 528216, China; 18702030877@163.com; 2 Institute of Environmental Pollution and Health, School of Environmental and Chemical Engineering, Shanghai University, Shanghai 200444, China

**Keywords:** organophosphate esters, private car microenvironments, indoor air, human exposure

## Abstract

Limited research has characterized the occurrence of organophosphate esters (OPEs) in indoor microenvironment air. To address this gap, ten OPE congeners were measured in air samples collected from 46 homes, 12 offices, 6 student dormitories, and 60 private cars in Guangzhou, China. Among the four microenvironments, private vehicles exhibited the highest total OPE concentrations (ΣOPEs), with an average of 264.89 ng/m^3^—statistically significantly higher than the other three environments (*p* < 0.05). This finding underscores the need for increased attention to OPE environmental fate in vehicles and associated human exposure risks. Distinct compositional profiles of OPEs were observed across microenvironments. In homes, offices, and student dormitories, tris(2-chloroethyl) phosphate (TCEP) and tris(2-chloropropyl) phosphate (TCPP) dominated the OPE mixture, accounting for 56% and 34% of ΣOPEs, respectively. By contrast, private cars were characterized by elevated levels of TCPP (68% of ΣOPEs) and tris(1,3-dichloro-2-propyl) phosphate (TDCP, 12%), reflecting source-specific emission patterns related to automotive materials. Significant correlations existed in most of the OPEs in the private cars, indicating that there are many potential sources of OPEs in private cars, and one source may release multiple OPEs. Human inhalation exposure to OPEs was estimated based on measured air concentrations. Daily respiratory exposure doses ranged from 9.1 to 30.85 ng/kg/d across different populations, with all values falling below established thresholds for non-carcinogenic and carcinogenic risks. These results indicate that current indoor air OPE levels in the studied microenvironments do not pose significant health hazards via inhalation pathways under typical exposure scenarios.

## 1. Introduction

Organophosphate esters (OPEs) represent a class of synthetic industrial additives widely employed as flame retardants and plasticizers in diverse applications, including furniture, electrical/electronic devices, textiles, and automotive components. After the restriction and phase-out of brominated flame retardants such as polybrominated diphenyl ethers (PBDEs) and hexabromocyclododecane (HBCD), OPEs are the main alternative flame retardants [1]. However, accumulating evidence indicates that certain OPE congeners have the characteristics of persistent organic pollutants (POPs): high toxicity, persistence, bioaccumulation, and long-range transport [2]. Similar to PBDEs and HBCD, OPEs are used additively in the materials, which means that they are not fixed in the polymer product through chemical binding and could easily leak into the environment via self-volatilization and product wear during the use of materials, causing potential hazards to the ecological environment and human health. Currently, OPEs have been detected in a broad spectrum of environmental matrices worldwide, including air [3], water [4,5], soil [6], sediment [7], aquatic biota (e.g., fish) [8], and human biomonitoring samples (e.g., breast milk) [9]. Regarding health effects, a recent review has concluded that organophosphate esters (OPEs) are rapidly absorbed after oral intake and inhalation, and can induce hazards such as oxidative stress, inflammatory responses, and endocrine disruption in organisms [10].

Human exposure risk to OPEs is significantly elevated in indoor microenvironments compared to outdoor settings, attributable to two primary factors. Firstly, modern urban residents typically spend over 70% of their time in indoor spaces, increasing prolonged contact with OPE-laden materials. Secondly, analytical data consistently demonstrate substantially higher OPE concentrations in indoor air (often orders of magnitude greater than outdoor air), driven by continuous emissions from building materials and consumer products. OPEs are intentionally incorporated into up to 5% of common indoor materials—such as polyurethane foams, plastic composites, and textile coatings—where they exist as non-covalently bound additives. These material matrices serve as significant reservoirs and continuous sources of releases of OPEs in the indoor environment. Wang et al. [11] reported that target OPEs were ubiquitously detected in the indoor air of 16 Beijing households, with total OPE concentrations (ΣOPEs) exceeding ΣPBDEs by at least an order of magnitude. In a Norwegian residential study, Kucharska et al. [12] observed distinct distribution patterns between air and dust matrices: tris(2-chloropropyl) phosphate (TCPP) dominated indoor air with a median concentration of 128 ng/m^3^, while tris(2-butoxyethyl) phosphate (TBEP) exhibited higher abundance in dust samples, reaching a maximum of 8100 ng/g. Additionally, Sun et al. [13] monitored the change in OPE concentration in the indoor air of a household in Harbin, China, over a year, and found that the concentrations of OPEs were higher in the spring and summer than in the autumn and winter.

Indoor contamination of OPEs in public spaces warrants equal attention. Kim et al. [14] analyzed OPEs in ten U.S. indoor microenvironments and recorded the highest average OPE concentration of 258 ng/m^3^ in auto parts stores, followed by electronic stores, nail salons, and furniture stores (in descending order of contamination). In a Swedish study, Bergh et al. [15] investigated air and dust samples from three Stockholm microenvironments—residential homes, day-care centers, and offices—where nine OPE congeners were ubiquitously detected. Day-care centers exhibited the highest air pollution levels, surpassing offices and homes, indicating elevated exposure risks for young children in these high-activity environments. Wang et al. [11] reported varying OPE concentrations across Beijing microenvironments: activity rooms (14.4 ng/m^3^) and student dormitories (19.4 ng/m^3^) had lower levels compared to offices (29 ng/m^3^) and family homes (24 ng/m^3^), reflecting source contributions from office equipment and consumer products. In addition, some studies have shown that OPE concentrations in indoor environments correlate with internal exposure levels in humans, e.g., positive correlations have been identified between air/dust OPE concentrations and their presence in human hair [12], while urinary metabolites of OPEs demonstrate strong associations with parent compound levels in indoor dust or hand towel samples [16]. These findings underscore the role of indoor microenvironments as critical pathways for OPE uptake.

The automobile interior represents another critical indoor microenvironment with pronounced OPE contamination. Although daily exposure duration in vehicles is generally shorter than in residential or office settings, OPE concentrations measured within car compartments frequently exceed those of other indoor environments. He et al. [17] analyzed dust samples from offices, public spaces, and automobiles in Nanjing, reporting the highest OPE levels in vehicle dust—16-fold and 6-fold greater than office and public microenvironment samples, respectively. Ali et al. [18] observed similar trends in Kuwaiti and Pakistani vehicles, where OPE concentrations in automobile dust were approximately five times higher than in residential or workplace dust matrices. Brommer et al. [19] compared OPE levels in dust from UK automobiles, school classrooms, homes, and offices, identifying tris(1,3-dichloro-2-propyl) phosphate (TDCIPP) as the dominant congener in vehicles with significantly elevated concentrations relative to other microenvironments. Brandsma et al. [20] further reported TDCIPP in car seat dust at 1100 μg/g—the highest concentration documented in the literature to date—highlighting automotive interiors as hotspots for OPE accumulation. While existing research confirms elevated OPE pollution in vehicle compartments, current investigations are overwhelmingly focused on dust matrices, with limited data available on airborne concentrations and associated human exposure risks. A recent screening assessment demonstrated that partial dust removal interventions in vehicles fail to reduce human contact with OPEs, implying the existence of additional exposure pathways beyond dust-mediated routes [21]. These findings underscore the urgent need for a comprehensive evaluation of airborne OPE levels in private vehicles. As the average person spends over 200 h per year in vehicles, understanding the exposure risks in this confined microenvironment is crucial for developing effective mitigation strategies. In this study, passive air sampling was employed to collect air samples from four distinct indoor microenvironments—residential homes, offices, student dormitories, and private vehicles. This method offers several advantages over traditional active sampling techniques, including lower cost, longer sampling duration, and reduced disturbance to the indoor environment. The research aims to systematically investigate the concentration profiles and pollution characteristics of OPEs across these environments, characterize their potential emission sources, and assess the human exposure risk.

## 2. Materials and Methods

### 2.1. Sampling Collection

From September to October 2020, a total of 124 indoor air sampling sites were selected in Guangzhou, China, including 46 homes, 12 offices, 6 student dormitories, and 60 private cars (gasoline-powered, 3 to 6 years old), to characterize the occurrence and compositional profiles of OPEs in diverse microenvironments. At each sampling site, at least one polyurethane foam-based passive air sampler (PUF-PAS) was deployed at 0.5–1.5m height for about 30 consecutive days. The PUF disks (14 cm diameter, 13.5 cm thickness, 0.017 g/cm^3^ density, 200 cm^3^ volume) followed the standardized design described in Li et al. [22], with detailed sampling protocols available in these references and Supporting Information (Text S1). Post-sampling, PUF disks were immediately sealed in brown glass bottles and stored at −20 °C to prevent analyte degradation until chemical analysis. Sampling rates for target OPEs were derived from a prior Northern China regional study [23], with individual compound-specific rates ranging from 0.59 to 2.01 m^3^/d. These values account for environmental variability in temperature, humidity, and air turbulence, ensuring reliable exposure metric calculations.

### 2.2. Chemicals and Materials

Ten organophosphate ester (OPE) reference standards were acquired from AccuStandard (New Haven, CT, USA), including tri-n-butyl phosphate (TBP, 99%), tris(2-chloroethyl) phosphate (TCEP), tris(1-chloro-2-propyl) phosphate (TCPP), tris(1,3-dichloro-2-propyl) phosphate (TDCPP), triphenyl phosphate (TPEP), 2-ethylhexyl diphenyl phosphate (DPEHP), triphenyl phosphate (TPHP), tris(2-butoxyethyl) phosphate (TBEP), tris(2-ethylhexyl) phosphate (TEHP), and tricresyl phosphate (TCP). Surrogate standards (D15-TPHP and d27-TBEP) and an internal standard (d27-TnBP) were sourced from C/D/N Isotopes Inc. (Quebec, QC, Canada) and Cambridge Isotope Laboratories (Cambridge, MA, USA), respectively. All solvents—n-hexane, dichloromethane, acetone, methanol, acetonitrile, and ethyl acetate—were of pesticide-grade purity (Sigma-Aldrich, Taufkirchen, Germany). Analytical-grade silica gel (60–200 μm) and sodium sulfate were purchased from Sinopharm Group Co. Ltd. (Beijing, China). Prior to use, silica gel and sodium sulfate were heated at 450 °C for 4 h to remove organic contaminants and activated under anhydrous conditions.

### 2.3. Sample Preparation and Analysis

Detailed protocols for air sample extraction and cleanup have been described previously [24]. Briefly, PUF disks were spiked with known quantities of surrogate standards (D15-TPHP and d27-TBEP) and subjected to Soxhlet extraction for 48 h using 300 mL of a n-hexane/dichloromethane (1:1, *v*/*v*) mixture. Extracts were concentrated to 1 mL via rotary evaporation and solvent-exchanged to hexane. The concentrated samples were purified using silica gel columns: 25 mL of ethyl acetate was employed as the eluant to fractionate target analytes. Eluates containing OPEs were further reduced to 200 μL under a gentle nitrogen stream, after which a defined amount of internal standard (d27-TnBP) was added prior to instrumental analysis. Sample quantification was performed using an Agilent 6890N gas chromatograph coupled to a 5973 mass spectrometer (GC-MS), with high-purity helium as the carrier gas. Separation was achieved on a DB-5MS capillary column (30 m × 0.25 mm i.d., 0.25 μm film thickness) under electron ionization (EI+) selected ion monitoring (SIM) mode. Detailed information on quantification ions, verification ions, and instrument parameters is provided in the Supplementary Information (SI).

### 2.4. Quality Assurance/Quality Control

To minimize blank contamination, a series of analytical precautions was implemented. All glassware was baked at 450 °C for 4 h and rinsed with n-hexane and ethyl acetate prior to use. Additionally, all samples and glassware were covered with aluminum foil to prevent dust contamination. A procedural blank was analyzed alongside each batch of 5 samples to evaluate potential contamination. Instrumental Detection Limit (IDL) was determined by injecting low-concentration target analytes with a signal-to-noise ratio of no less than 3:1. The Method Detection Limit (MDL) was calculated as the mean blank sample concentration plus three times the standard deviation. When target compounds were not detected in blanks, half of the IDL was used as the MDL. In this study, the MDLs of OPEs in indoor air samples ranged from 0.022 to 1.21 ng/m^3^. The recovery efficiency of the procedure was verified by analyzing uncontaminated PUF spiked with OPE standards. The overall recovery was generally greater than 68%. The recovery rates of the surrogate standards ranged from 76% to 104% for D_27_—TBEP, and from 63% to 109% for D_15_—TPHP, respectively. No sample was corrected for recovery.

### 2.5. Exposure and Risk Assessment

The estimated daily intake (EDI) by inhalation of OPEs was estimated using Equation (1):

*EDI* = *C* × *IR* × *IEF*/*BW* (1)

where EDI is the estimated daily intake of each OPE through atmosphere inhalation (ng/kg/day); C is the concentration of OPEs in indoor ambient air (ng/m^3^); IR is the respiration rate (m^3^/day); IEF is the fraction of individual’s daily exposure; BW is the body weight of human (kg). We assumed 100% absorption of inhalation in the absence of experimental data for this parameter. IR and BW for Chinese were obtained from the report “Risk Assessment Guidance for Industrial Contaminated Sites in China”. The exposure assessment parameters for different age groups can be found in Table S2. The EDI of each OPE values was calculated based on a Monte Carlo simulation, which was performed using Crystal Ball software (Oracle, Austin, TX, USA.version:11.1.3.0.0) with independent runs of 1000 trials.

The non-carcinogenic hazard quotient (HQ) and carcinogenic risk (CR) index of inhaling OPEs were estimated using Equations (2) and (3), respectively:

*HQ* = *EDI*/*RfD* (2)

*CR* = *EDI* × *CSF* (3)

where HQ is the hazard quotient, EDI is the estimated daily intake of each OPE (ng/kg/day), RfD is the reference doses values for each OPEs (ng/kg bw/day), and CSF is the carcinogenic effect slope factor for each OPEs (mg/kg bw/day). Assuming that residents of Guangzhou are exposed for 24 h per day. HQ > 1 suggests that there is a potential health risk, whereas CR > 1 × 10^−6^ indicates potential adverse effects.

### 2.6. Data Analysis

Statistical analyses of air sample data were performed using SPSS version 19.0 (SPSS Inc., Chicago, IL, USA). Differences in airborne organophosphate ester (OPE) concentrations across microenvironments were evaluated using independent-samples *t*-tests to assess statistical significance. Spearman’s rank correlation analysis was employed to quantify the strength of associations between individual OPE congeners, with statistical significance defined as a *p* value < 0.05. Estimated Daily Intakes (EDIs) for individual OPEs were derived from Monte Carlo simulations implemented in Crystal Ball software, which generated probabilistic exposure distributions by incorporating input parameters such as air concentrations, breathing rates, and exposure durations. This approach accounts for variability in human activity patterns and microenvironmental conditions, providing robust estimates of potential human exposure.

## 3. Results and Discussion

**Figure 1:**
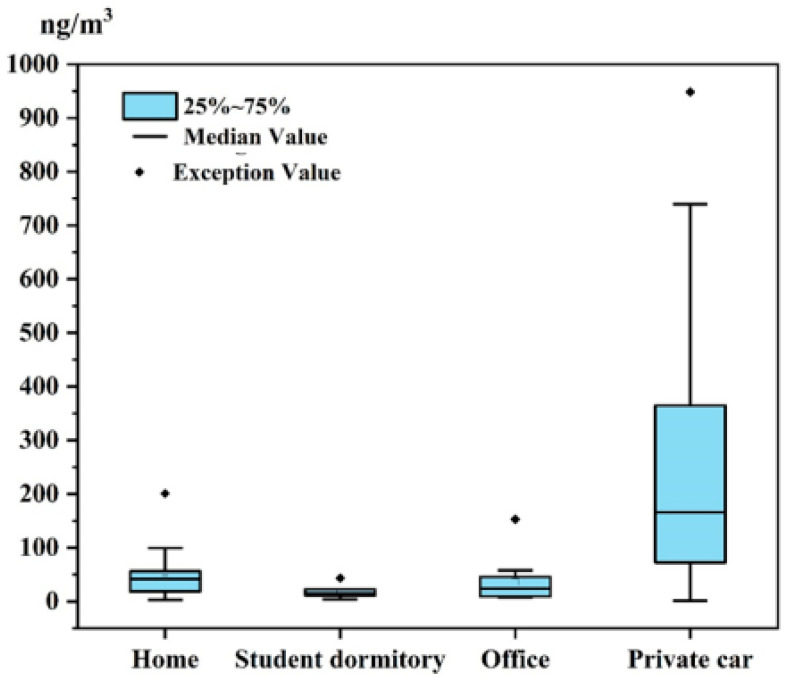
Total concentrations of OPEs in indoor air from different microenvironments.

Concentrations of ten OPEs were measured in indoor air samples collected from 60 private cars, 46 homes, 12 offices, and 6 student dormitories in Guangzhou, China. OPEs were ubiquitously detected across all four microenvironments. Average total OPE concentrations (ΣOPEs) in private car air (264.89 ng/m^3^) were significantly higher than those in homes (42.12 ng/m^3^), offices (36.02 ng/m^3^), and student dormitories (18.14 ng/m^3^), as determined by one-way ANOVA (*p* < 0.05; Figure 1). No statistically significant differences were observed in ΣOPEs levels among homes, offices, and dormitories. Given this pronounced disparity, the unique contamination profile of private car interiors is discussed separately in the subsequent analysis.

### 3.1. Total Concentrations and Profiles of OPEs in Indoor Air from China Homes, Offices, and Student Dormitories

All the target flame retardants (FRs) were detected in at least one of the indoor air samples, which implies the extensive use of a wide variety of OPEs in the study area. Table 1 presents the summary statistics and detection frequencies (DF) of OPEs in indoor air collected from homes, offices, and student dormitories in Guangzhou, China. TCEP, TCPP, DPEHP, TPHP, and TDCPP were the five most prevalent contaminants, with detection rates of 100%, 100%, 98.4%, 98.4%, and 88.7%, respectively. TEHP, TBP, and TBEP followed, with detection rates of 62.9%, 45.2%, and 25.8%, respectively. Given that the detection rates of TCP and TPEP were less than 3%, these two compounds were not further discussed. The total concentration of eight OPEs (Σ_8_OPEs) in indoor microenvironment air ranged from 2.99 to 200.76 ng/m^3^, with an average of 38.62 ng/m^3^ and a median of 32.78 ng/m^3^. Generally, the OPE concentrations in the indoor air of Guangzhou were higher than those previously reported for outdoor air [25]. This suggests that indoor environments serve as the primary source of OPEs to the outdoor environment, due to emissions from household products and materials used indoors. These results are consistent with previous research findings. In homes, offices, and student dormitories, the overall OPE concentration trend followed home > office > student dormitory, although statistical analysis revealed no significant differences among the three (*p* > 0.05). This finding aligns with previous observations of similar concentration patterns in Dalian residential microenvironments [26]. The relatively low Σ_8_OPE levels in student dormitories can be attributed to their simpler interior finishes and fewer consumer products, which likely reduce OPE emission sources. Available data on indoor air OPE concentrations from global studies are summarized in Table S3. Compared to these datasets, the average OPE concentration in Guangzhou indoor air was lower than reported values from Germany (81.89 ng/m^3^) [27], the United States (101 ng/m^3^) [13], and Sweden (160 ng/m^3^) [28]. Conversely, it was significantly higher than levels measured in India (0.483 ng/m^3^) [29], Egypt (0.007–0.064 ng/m^3^) [30], and Dalian, China (14.9 ng/m^3^) [26], while comparable to results from Australia (44 ng/m^3^) [31] and Canada (46 ng/m^3^) [32]. These regional disparities suggest a strong correlation between indoor OPE levels and the degree of urbanization/industrialization, with more developed areas generally exhibiting higher contamination, likely due to intensive use of OPE-containing materials and products.

**Figure 2:**
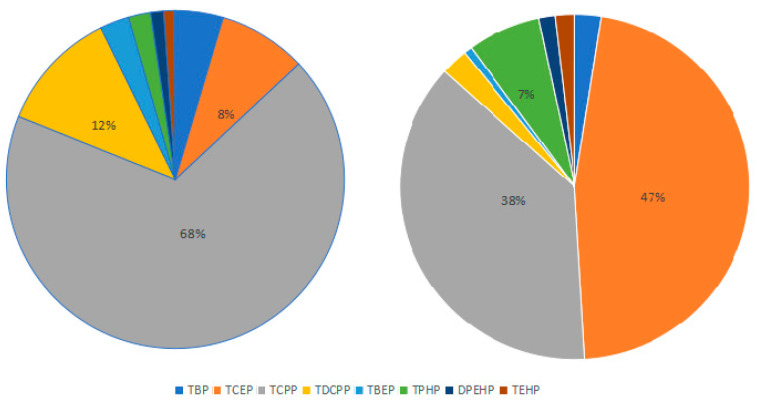
Distribution characteristics of OPEs in indoor air from different microenvironments. (**Left**): homes, offices, and student dormitories as a whole; (**Right**): Private car.

Among the analyzed compounds, tris(2-chloroethyl) phosphate (TCEP) was the most abundant OPE congener, with a mean concentration of 18.01 ng/m^3^, followed by tris(2-chloropropyl) phosphate (TCPP, 14.52 ng/m^3^) and triphenyl phosphate (TPHP, 2.61 ng/m^3^). The compositional profile of indoor air OPEs followed the order: TCEP (46.6%) > TCPP (37.6%) > TPHP (6.8%) > TDCPP (2.5%) > TBP (2.4%) > TEHP (1.8%) > DPEHP (1.5%) > TBEP (0.8%) (Figure 2, Left). Chlorinated OPEs dominated the mixture, accounting for 84.2% of total concentrations, with TCEP and TCPP as the primary homologs—consistent with findings from Lai et al. in Northern Chinese atmospheric fine particles [33]. Notable compositional differences were observed compared to global studies: TCPP and triethyl phosphate (TEP) accounted for 43% and 33% of U.S. air samples [14], while TCPP, triisobutyl phosphate (TiBP), and tri-n-butyl phosphate (TnBP) represented 45%, 28%, and 12% in German air [27]. In Dalian, China, TCPP (74%), TBP (12%), and TDCPP (5%) dominated the profile [26]. These disparities reflect regional regulatory and usage patterns: since 2016, the U.S. and EU have restricted TCEP due to its neurotoxic and carcinogenic properties, leading to increased reliance on TCPP as a substitute—explaining its elevated presence in those regions. In this study, the high abundance of TCEP and TCPP can be attributed to multiple factors: their relatively high saturated vapor pressures facilitate volatilization from materials, combined with environmental persistence due to slow degradation rates. Additionally, their historical widespread use in China for flame retardancy in plastics, foams, and textiles likely contributes to their dominant presence in indoor air.

### 3.2. Total Concentrations and Profiles of OPEs in Indoor Air of Private Cars from China

Table 2 presents the concentration profiles of OPEs in private vehicle air. All target OPEs were detected in at least one sample, demonstrating their universal presence in car interiors. Tris(2-chloropropyl) phosphate (TCPP) was the only congener with a 100% detection rate, followed by TCEP, TDCPP, and TPHP (detection rates > 90%). Moderate detection rates were observed for TBP (75%), DPEHP (73.7%), and TEHP (71.7%), while tBEP (21.7%) and TPEP (11.7%) exhibited lower detection frequencies. TCP was detected in only 3 out of 60 vehicles and was excluded from further analysis. Total OPE concentrations (Σ_9_OPEs) in private car air exhibited substantial variability, ranging from 1.22 to 1288.81 ng/m^3^—a 1000-fold difference between minimum and maximum values. TCPP dominated the OPE profile with a mean concentration of 180.33 ng/m^3^, significantly higher than all other congeners (*p* < 0.001). The highest individual TCPP concentration reached 1087.38 ng/m^3^, reflecting diverse emission sources in vehicle interiors. This dominance is likely attributed to TCPP’s widespread use as a flame retardant in polyvinyl chloride (PVC) and polyurethane foam materials commonly found in automotive upholstery and dashboard components [34]. Global comparisons (Table S4) highlight limited airborne OPE research in vehicle microenvironments. Swedish vehicle air reported an average ΣOPEs of 2065 ng/m^3^—approximately eight times higher than this study [28]. In Japan’s Yokohama region, although most OPEs were undetected or below limits, TCPP reached a maximum of 1500 ng/m^3^, comparable to the highest TCPP concentration observed here (1087 ng/m^3^) [35]. Kim et al. found U.S. private car air averaged 59 ng/m^3^, with auto parts stores exhibiting higher levels (258 ng/m^3^), consistent with OPE use in automotive plastics and flame retardants [14]. German studies in the Rhine region reported ΣOPEs ranging from 26.18 to 751 ng/m^3^ (mean: 265.9 ng/m^3^) [27], while Swiss measurements in Zurich recorded a TCPP mean of 260 ng/m^3^ [36]. Collectively, these data indicate that OPE concentrations in this study fall within the moderate range compared to global vehicle microenvironments.

The compositional profile of OPEs in private vehicle air (Figure 2, Right) followed the order: TCPP (68.1%) > TDCPP (11.6%) > TCEP (8.4%) > TBP (4.6%) > TBEP (2.8%) > TPHP (2.1%) > DPEHP (1.2%) > TEHP (1.0%) > TPEP (0.1%). Chlorinated OPEs—TCPP, TDCPP, and TCEP—dominated the mixture, accounting for 88.2% of total concentrations, with TCPP as the primary contaminant. Non-chlorinated alkyl esters (TBP, TBEP, TEHP, TPEP) contributed 8.5% of the total OPEs load. This composition closely resembled that reported for German Rhine-region private cars, where chlorinated OPEs accounted for 60% of total concentrations [27]. Notably, TCPP represented 86% of total chlorinated OPEs in this study, with TDCPP comprising 14%, while TCEP was undetected in German samples—likely due to regional restrictions on TCEP production and use. Consistent with findings from Sweden, the U.S., Switzerland, and Japan, TCPP emerged as the dominant congener in transportation microenvironments globally [14,28,35,36]. This compositional similarity suggests shared emission sources across different regions, primarily the use of chlorinated OPEs (e.g., TCPP, TDCPP) as flame retardants in flexible polyurethane foams and polyvinyl chloride materials commonly used in automotive interiors. Additional influencing factors include vehicle service life, manufacturing origins, and regional regulatory policies on specific OPE congeners. Correlation analysis among seven OPEs (excluding TBEP and TPEP due to low detection rates) revealed significant associations, see Table S5. TPHP correlated strongly with five congeners (*p* < 0.05), except for a weak relationship with TBP (r = 0.188, *p* > 0.05), likely due to shared physicochemical properties with DPEHP/TEHP, indicating common sources. TCPP exhibited significant positive correlations with TCEP (r = 0.401, *p* < 0.01) and TDCPP (r = 0.546, *p* < 0.001), consistent with co-use in industrial formulations. While most OPE pairs showed significant correlations, moderate correlation coefficients (r < 0.6) suggest contributions from multiple emission pathways—such as in-car air exchange, occupant-introduced contaminants, or differential environmental behaviors (e.g., volatility, degradation rates) of individual congeners. These findings highlight the complex interplay between source inputs and microenvironmental processes in shaping OPE distribution patterns.

### 3.3. Exposure Levels and Health Risks of OPEs in Four Indoor Environments for Different Populations

In this study, the Monte Carlo algorithm was employed to calculate the daily respiratory exposure doses of different populations, using the total indoor air concentrations of OPEs measured in homes, offices, student dormitories, and private cars in Guangzhou. The results are presented in Table 3. Infants exhibited the highest exposure level, with a mean daily exposure of 30.85 ng/kg/day, followed by toddlers and children, whose mean daily exposures were 15.89 ng/kg/day and 12.41 ng·kg^−1^·d^−1^, respectively. Adolescents and adults had lower daily respiratory exposures compared to infants, toddlers, and children. A general trend of decreasing daily OPE exposure levels in indoor ambient air with increasing age was observed among different populations. When compared with other studies, the mean daily exposures of young children and adults to indoor air OPEs in homes in Beijing were 2.1 ng/kg/day and 1.2 ng/kg/day, respectively [37]. In Australia, the daily respiratory exposures of young children and adults to indoor air OPEs were 7 ng/kg/day and 7.9 ng/kg/day [31], both of which were lower than those of the corresponding age groups in this study. The relatively high exposure levels of OPEs in the indoor ambient air observed in this study might be attributed to the elevated OPE concentrations detected in the microenvironment of private cars.

**Figure 3:**
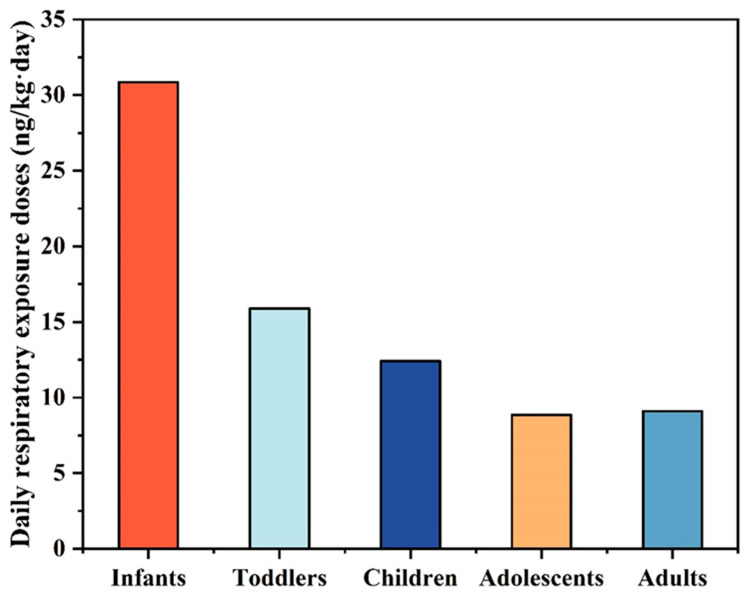
Comparison of the average daily respiratory exposure of OPEs to different populations.

Sensitivity analyses were conducted to investigate the impacts of parameters, including the concentration of OPEs in various indoor environments (C), respiration rate (IR), and body weight (BW), on the respiratory exposure doses of different populations. The contribution of each factor to the estimated daily intake (EDI) was calculated, and the results are detailed in Table S6 and Figure 3. The contribution ratios of IR and BW to the infant exposure dose (approximately 10%) were higher than those for other populations. This indicates that infants are more sensitive to OPE exposure, likely due to their unique crawling posture and lower body weight compared with the other four populations. For infants and toddlers, only their exposure in the home environment was considered. The concentration of OPEs in home air contributed 93–99.5% and 75.3–98.6% to the exposure of toddlers and infants, respectively. For children and adolescents, the OPE concentration in private cars was the primary contributor to their daily respiratory exposure doses (children: 60.2%; adolescents: 61.6%), followed by the influence of the study office environment (children: 22.6%; adolescents: 23.1%). For adults, the OPE concentration in homes was the main contributor to their daily respiratory exposure doses (50.7%), with private cars following as the second major contributor (30.3%).

The non-carcinogenic risk quotient (HQ) and carcinogenic risk index (CR) were employed to assess the respiratory exposure risks of OPEs in different indoor air environments for residents of Guangzhou City, as presented in Table 4. The table shows that the mean HQ values for different populations in this region were much less than one, suggesting that the current exposure levels of OPEs in various indoor environments in Guangzhou do not pose non-carcinogenic risks to humans. Carcinogenic risk assessment results indicated that the CR values for infants, toddlers, children, adolescents, and adults were all below 1 × 10^−6^, indicating that the OPE exposure levels in different indoor environments in Guangzhou City did not present carcinogenic risks in this study. However, it is noteworthy that the mean carcinogenic effect value of OPEs for infants was 0.33 × 10^−6^, the highest among the five age groups. Considering that OPEs can enter the human body not only through respiratory exposure but also via dermal contact, dust ingestion, and diet, and that infants have more frequent dermal contact and dust ingestion routes compared with the other four age groups, potential health risks for infants may still persist.

## 4. Conclusions

This study investigated the concentration levels and compositional characteristics of OPEs in 124 air samples from four indoor microenvironments in Guangzhou, China—households, offices, student dormitories, and private cars—and evaluated human respiratory exposure risks. Results showed that organophosphorus flame retardants are ubiquitous organic pollutants in Guangzhou’s indoor air, with concentrations falling within the global low-to-medium pollution range. Significantly higher ∑OPEs (sum of OPEs) concentrations were observed in private car indoor air compared to the other three microenvironments, highlighting the need for increased attention to OPE environmental behavior in vehicles and associated human exposure risks. Respiratory exposure risk assessments indicated low inhalation-based exposure levels of OPEs for all populations in Guangzhou, with no significant overall non-carcinogenic or carcinogenic health risks identified. Nevertheless, this study also has certain limitations. In terms of spatial and temporal scales, the research only selected four types of micro-environments in Guangzhou, without considering climate, ventilation design, and industrial differences, and did not cover seasonal variations. Therefore, it is difficult to reflect the temporal and spatial dynamics of OPE concentrations. The pollution source analysis is not in-depth, there is no direct traceability of materials, and no detection of emerging OPEs. In terms of exposure pathways, only respiratory exposure was evaluated, without considering dermal contact, dietary intake, and dust contact, especially the multi-pathway exposure situations of special groups such as infants, which may underestimate the actual risk. Future research can be conducted in the directions of multi-media sampling, cross-regional seasonal studies, and material traceability.

## Figures and Tables

**Table 1:** Summary statistics and detection frequencies (DF) of OPEs in indoor air collected from homes, offices, and student dormitories in Guangzhou, China (ng/m^3^).

Compounds	DF (%)	Mean	Percentile					
			Min	25th	50th	75th	90th	Max
TBP	45.2	0.95	ND	<MDL	<MDL	1.02	2.59	16.15
TCEP	100	18.01	0.27	2.16	11.71	30.92	42.65	92.20
TCPP	100	14.52	0.10	3.74	7.26	13.90	36.26	108.36
TDCPP	88.7	0.96	ND	0.67	0.85	1.11	1.61	6.86
TBEP	25.8	0.29	ND	<MDL	<MDL	0.50	1.01	3.39
TPHP	98.4	2.61	ND	0.31	1.26	2.51	6.06	43.18
DPEHP	98.4	0.58	ND	0.26	0.33	0.40	0.94	11.51
TEHP	62.9	0.70	ND	<MDL	0.36	0.52	1.84	8.79
Σ_8_OPEs	100	38.62	2.99	14.88	32.78	52.11	71.12	200.76

DF: detectable frequencies; ND: not detected. MDL: Method Detection Limit.

**Table 2:** Summary statistics and detection frequencies (DF) of OPEs in indoor air collected from private cars in Guangzhou, China (ng/m^3^).

Compounds	DF (%)	Mean	Percentile					
			Min	25th	50th	75th	90th	Max
TBP	75%	12.12	ND	0.0076	3.68	12.95	37.73	89.6
TCEP	95%	22.36	ND	9.25	14.04	33.24	47.82	105.99
TCPP	100%	180.33	0.06	17.78	75.52	242.96	540.17	1087.38
TDCPP	91.7%	30.84	ND	1.19	3.66	7.05	15.00	827.49
TBEP	21.7%	7.36	ND	<MDL	<MDL	<MDL	38.60	72.86
TPHP	93.3%	5.65	ND	0.64	1.85	5.07	10.03	108.8
DPEHP	73.7%	3.30	ND	<MDL	0.83	8.60	8.87	22.94
TEHP	71.7%	2.61	ND	<MDL	0.87	2.99	4.16	70.21
TPEP	11.7%	0.31	ND	<MDL	<MDL	<MDL	1.25	3.81
Σ_9_OPEs	100%	264.89	1.22	71.79	165.58	369.65	702.25	1288.81

DF: detectable frequencies; ND: not detected. MDL: Method Detection Limit.

**Table 3:** Daily respiratory exposure dose (ng/kg/day) of OPEs in indoor air of different age groups.

Infants	TBP	TCEP	TCPP	TDCPP	TPHP	DPEHP	TEHP	ΣOPEs
Mean	0.73	15.13	11.26	0.76	0.29	2.35	0.5	30.85
P5	0.02	2.78	1.15	0.16	0	0.75	0.02	7.42
P50	0.26	10.66	6.32	0.51	0.01	1.97	0.18	23.15
P95	2.71	42.13	37.08	1.62	0.74	5.17	1.96	79.18
Toddlers	TBP	TCEP	TCPP	TDCPP	TPHP	DPEHP	TEHP	ΣOPEs
Mean	0.39	7.77	5.61	0.38	0.15	1.19	0.27	15.89
P5	0.01	1.54	0.63	0.09	0	0.44	0.01	4.25
P50	0.14	5.61	3.32	0.27	0.01	1.03	0.09	12.05
P95	1.45	21.09	17.85	0.77	0.41	2.48	1.02	39.91
Children	TBP	TCEP	TCPP	TDCPP	TPHP	DPEHP	TEHP	ΣOPEs
Mean	0.45	2.69	7.55	0.94	0.44	0.17	0.13	12.41
P5	0.09	0.97	2.08	0.23	0.15	0.08	0.02	4.74
P50	0.31	2.24	5.79	0.39	0.35	0.13	0.08	10.34
P95	1.25	5.94	18.92	1.66	0.96	0.36	0.35	27.39
Adolescents	TBP	TCEP	TCPP	TDCPP	TPHP	DPEHP	TEHP	ΣOPEs
Mean	0.32	1.94	5.55	0.66	0.31	0.12	0.09	8.86
P5	0.06	0.71	1.53	0.17	0.11	0.06	0.02	3.43
P50	0.22	1.6	4.2	0.28	0.25	0.1	0.06	7.37
P95	0.86	4.26	13.6	1.18	0.68	0.26	0.25	19.12
Adults	TBP	TCEP	TCPP	TDCPP	TPHP	DPEHP	TEHP	ΣOPEs
Mean	0.29	3.37	4.27	0.48	0.18	0.39	0.13	9.1
P5	0.06	1.16	1.09	0.1	0.04	0.16	0.02	3.63
P50	0.2	2.78	3.28	0.24	0.12	0.34	0.08	7.9
P95	0.8	7.51	10.69	0.81	0.46	0.76	0.39	18.59

**Table 4:** Health risks of OPEs in indoor air of Guangzhou to different populations.

Compounds	Non-Carcinogenic Risk Quotient ^a^ (×10^−4^)					Carcinogenic Risk Index ^b^ (×10^−6^)				
	Infants	Toddlers	Children	Adolescents	Adults	Infants	Toddlers	Children	Adolescents	Adults
TBP	0.73	0.39	0.45	0.32	0.29	0.0066	0.0035	0.0041	0.0029	0.0026
TCEP	21.61	11.10	3.84	2.77	4.81	0.30	0.16	0.054	0.039	0.067
TCPP	11.26	5.61	7.71	5.55	4.27	-	-	-	-	-
TDCPP	0.38	0.19	0.47	0.33	0.24	0.024	0.012	0.029	0.020	0.015
TPHP	0.041	0.021	0.063	0.044	0.026	-	-	-	-	-
TEHP	0.050	0.027	0.013	0.009	0.013	0.002	0.001	0.000	0.000	0.000
Σ_6_OPEs	34.08	17.34	12.55	9.02	9.65	0.33	0.17	0.087	0.062	0.085

^a^ Reference dose (ng·kg^−1^ bw day^−1^), data from United States Environmental Protection Agency. The values of TBP, TCEP, TCPP, TDCPP, TPHP, and TEHP are 10,000, 7000, 10,000, 20,000, 70,000, and 100,000, respectively. ^b^ Cancer slope factor ((mg·kg^−1^ bw day^−1^)^−1^), data from United States Environmental Protection Agency. The values of TBP, TCEP, TDCPP, and TEHP are 0.009, 0.02, 0.031, and 0.0032, respectively.

## Data Availability

The data that support the findings of this study are available from the corresponding author upon reasonable request.

## Supplementary Materials

The following supporting information can be downloaded at: https://www.mdpi.com/article/10.3390/toxics13070531/s1, Text S1: Passive Sampling Procedure; Text S2: Instrumental Analysis; Table S1: Retention time and qualitative and quantitative ions of OPEs; Table S2: Exposure assessment parameters of different age groups; Table S3: Comparison of ΣOPE concentrations (ng/m^3^) in indoor air around different areas in the world range (mean or mean range); Table S4: Concentration level of OPEs in the air of private cars (ng/m^3^); Table S5: Correlations between various OPEs in air of private cars; Table S6. Contribution rate (%) of different exposure parameters (C, IR, and BW) to the daily respiratory exposure of OPEs in different populations [14,26,27,28,29,30,31,35,36].
