# Supplementary material for: Concentrations, Compositions and Human Exposure Risks to Organophosphate Esters in Indoor Air from Various Microenvironments in Guangzhou, China
Source: Toxics. 2025 Jun 25;13(7):531. doi: 10.3390/toxics13070531 (PMC12300020; doi:10.3390/toxics13070531)
Supplement: Supplementary file 1 [file toxics-13-00531-s001.zip › toxics-3673725-supplementary.pdf]

Supporting Information

Concentrations, compositions and human exposure risks to organophosphate esters in indoor air from various microenvironments in Guangzhou, China

Maoyuan Xu [1,†], Yunmei Cai [2,†], Minghui Ouyang [1], Yusheng Wu [1], Ruijie Wang [1], Kewen Zheng [1] and Guofa Ren [1,*]

[1] Institute of Environmental Pollution and Health, School of Environmental and Chemical Engineering, Shanghai University, Shanghai 200444, China; 22722775xmy@shu.edu.cn (M.X.); ouyangminghui@shu.edu.cn (M.O.); 2365544895@shu.edu.cn (Y.W.); wangruijie@shu.edu.cn (R.W.); kewenzheng@shu.edu.cn (K.Z.)

[2] School of Environmental Monitoring, Guangdong Polytechnic of Environmental Protection Engineering, Foshan 528216, China; 18702030877@163.com

* Correspondence: rgf2008@shu.edu.cn; Tel./Fax: +86-21-66137753

† These authors contributed equally to this work.

List of Contents

- **Text S1.Passive Sampling Procedure**
- **Text S2. Instrumental Analysis**
- **Table S1. Exposure assessment parameters of different age groups**
- **Table S2. Comparison of ΣOPEs concentrations (ng/m^3) in indoor air around different areas in the world range (mean or mean range).**
- **Table S3. Comparison of ΣOPEs concentrations (ng/m^3) in the air of private cars (ng/m3) around different areas in the world range (mean or mean range).**
- **Table S4. Correlations between various OPEs in air of private cars.**
- **Table S5. Contribution rate (%) of different exposure parameters (C, IR and BW) to the daily respiratory exposure of OPEs in different populations.**

Passive Sampling Procedure.

Before sampling, make sure that the indoor places are maintained in daily use, and keep the doors and windows closed at all times during the sampling period. PUF discs used for sampling, radius 7cm, average thickness 13.5cm, volume of about 200cm^3, need to be dichloromethane and n-hexane (volume ratio of 1:1) mixture of continuous extraction solution for 72 hours before use, after the completion of the extraction of the vacuum drying, after the dry discs wrapped in aluminum foil, stored in a low-temperature environment at - 20 °C standby. PUF passive sampler consists of four parts: upper and lower stainless steel shell, central screw and PUF sponge disc， as shown in Fig. S1. For actual sampling, the PUF sponge discs are first placed in the lower housing and then covered with the larger upper housing, which is fastened by the central screw. The sampler is then suspended vertically under the roof of the room using the fixing clips of the upper housing. The target substances in the air are adsorbed into the PUF disk by diffusion, and the pollutants are collected. In order to minimize the interference of human activities with the sampling results, the passive air sampler should be placed horizontally and at a height of 0.5 - 1.5 meters above the floor.

Instrumental analysis.

The determination of OPEs was performed with an Agilent 6890N gas chromatograph (GC) coupled to an Agilent 5975 mass spectrometer (MS). The GC was fitted with a DB-XLB capillary column (30m, 250mm internal diameter, 0.25 mm film thickness, Agilent). Ultra-pure helium was used as carrier gas under constant flow mode at a flow rate of 1.2 ml/min; Manual Injections (1uL) were made in the pulse splitless mode; The injection temperature was set at 280°C. Initial column temperature was held at 80 °C for 1 min, and then programmed to 180 °C at 10 °C/min (held for 1 min), to 270 °C at 2 °C/min (held for 1 min), and to 300 °C at 15 °C/ min (held for 10 min). Mass spectrometry analysis conditions: Ionization mode was electron ionization, interface temperature was 230°C, electron energy was 70 eV, transfer line temperature was 280°C, and SIM scanning mode was adopted. The characteristic ions and retention times for qualitative determination of 12 target OPEs and deuterated standards are shown in the following table.

TableS1 Retention time and qualitative and quantitative ions of OPEs

Compounds	Retention time（min）	Qualitative ions（m/z）	Quantitative ions（m/z）
TEP	7.33	155	109，127
TBP	14.61	99	155，211
TCEP	16.35	63	249，251
TCPP	16.93	125	277，279
TPEP	19.62	169	169，239
TDCPP	30.96	75	191，381
TPHP	33.38	326	325，326
TBEP	34.34	125	199，299
DPEHP	34.5	251	251，362
TEHP	36.2	99	211，323
ΣTCP	42,43,44	368	367，368
d_{27}-TBP	14.38	167	167，231
d_{15}-TPHP	33.1	341	340，341
d_{21}-TBEP	33.66	126	126，209

Table S2. Exposure assessment parameters of different age groups

Population groups (year)	Indoor Exposure Fraction （IEF）	Body weight (BW, Kg)	Inhalation rate (IR, m3/d)	OPE concentration (ng/m3)e
Infants(1<)	0.799a	NDd （5,1.01）	NDd （4.5,1.01）	UDf (min, max)
Toddlers (1-5)	0.799a	NDd （16,1.02）	NDd （7.6,1.01）	UDf (min, max)
Children (6-11)	0.44a,0.27b,0.06c	NDd （29,1.04）	NDd （10.9,1.01）	UDf (min, max)
Adolescents （12-19）	0.44a,0.27b,0.06c	NDd （52,1.04）	NDd （14,1.01）	UDf (min, max)
Adults （≥20）	0.51a,0.29b,0.05c	NDd（65,1.14）	NDd（13.3,1.01）	UDf (min, max)

a: Day fraction of people within the home；b: Day fraction of people within the office；c: Day fraction of people within the vehicle; d: ND: normal distribution with geometric mean and geometric standard deviation: ND (gm,gsd); e: OPE concentration in this study; f: UD: uniform distribution with minimum and maximum: UD (min, max).

TableS3. Comparison of ΣOPEs concentrations (ng/m^3) in indoor air around different

areas in the world range (mean or mean range).

Sampling site	Sampling year	Compound description	ΣOPEs concentrations	reference
Dalian,China	2016.12-2017.5	9	1.65-103（mean:14.9）	Wang et al. (2021)
Bihar, Indian	2015.8-2015.10	8	0.897-2.16（mean:0.483）	Yadv et al. (2020)
Alexandria, Egypt	2014.11-2014.12	7	0.007-0.064 (-)	Khairy et al. (2019)
Brisbane and Canberra, Australia	2015.1-2015.3	9	7.2-760（median：44）	He et al. (2018)
Rhine, Germany	2015.1-2015.7	9	3.3-751（mean:81.89）	Zhou et al. (2017)
New York State, USA	2018.1	15	2.96-635（mean:101）	Kim et al. (2019)
Sweden, Stockholm	2016.12-2017.2	10	20-480（median：160）	Staaf et al. (2005)
Guangzhou,China	2020.9-2020.10	10	2.99-200.76（mean:38.62）	This study

Table S4.Comparison of ΣOPEs concentrations (ng/m^3) in the air of private cars (ng/m^3) around different areas in the world range (mean or mean range).

Sampling site	Sampling year	Compound description	ΣOPEs concentrations	reference
Stockholm, Sweden	N.R	10	median：2065	Staaf et al. (2005)
New York, USA	2018.1	15	mean: 59	Kim et al. (2019)
Yokohama, Japan	2013.12-20137.12	9	TCPP max: 1500	Tokumura et al. (2017)
Zurich, Switzerland	N.R	8	mean: 124	Tokumura et al. (2017)
Rhine, Germany	2015.1-2015.7	9	mean: 265.9	Zhou et al. (2017)
Guangzhou,China	2020.9-2020.10	10	mean: 264.89	This study

Table S5. Correlations between various OPEs in air of private cars

TBP	TCEP	TCPP	TDCPP	TPHP	DPEHP	TEHP
1						
0.306*	1					
0.304*	0.401**	1				
0.236	0.188	0.546**	1			
0.188	0.592**	0.331**	0.329*	1		
0.144	0.455**	0.211	0.347**	0.546**	1	
0.081	0.131	0.121	0.415**	0.313*	0.160	1

**.$p < 0.01$； *.$p < 0.05$

Table S6. Contribution rate (%) of different exposure parameters (C, IR and BW) to the daily respiratory exposure of OPEs in different populations

Infants	TBP	TCEP	TCPP	TDCPP	TPHP	DPEHP	TEHP	OPEs
C-J	95.9	88.7	92.9	88.9	98.6	75.3	96.2	86.2
IR	2.4	6.3	3.9	6.4	0.8	13.9	2.4	7.7
BW	1.7	5	3.2	4.7	0.6	10.8	1.4	6.1
Toddlers	TBP	TCEP	TCPP	TDCPP	TPHP	DPEHP	TEHP	OPEs
C-H	99.2	96.8	98.2	97.1	99.5	93	99.2	96.1
IR	0.5	2.3	1.5	2.4	0.5	5.8	0.6	3.1
BW	0.3	0.9	0.3	0.5	0	1.2	0.2	0.8
Children	TBP	TCEP	TCPP	TDCPP	TPHP	DPEHP	TEHP	OPEs
C-D	9	7	17.5	0.5	31.4	1.8	7	12.9
C-O	16.2	80.3	12.8	3.2	31.3	19.4	43.9	22.6
C-P	73.4	9.5	66.8	93.5	33.4	73.1	47.3	60.2
IR	1.2	2.9	2.6	2.4	3.5	4.8	1.7	4
BW	0.2	0.3	0.3	0.4	0.4	0.9	0.1	0.3
Adolescents	TBP	TCEP	TCPP	TDCPP	TPHP	DPEHP	TEHP	OPEs
C-D	9.3	7.4	17.7	0.3	32.2	2.5	7.8	12.8
C-O	15.5	80.5	13.7	2.5	32.9	19.6	45.2	23.1
C-P	74.3	9.8	66.2	95.7	32.7	74.4	45.9	61.6
IR	0.8	2.1	1.7	1.3	2.1	3.3	1	2.3
BW	0.1	0.2	0.7	0.2	0.1	0.2	0.1	0.2
Adults	TBP	TCEP	TCPP	TDCPP	TPHP	DPEHP	TEHP	OPEs
C-H	36.1	73.1	34.8	22.8	9.1	94.8	63.4	50.7
C-O	14.9	22.4	14.7	1.5	53.2	0.8	18.4	15.7
C-P	47.1	1.2	48.1	74.4	35.9	4.3	18.2	30.3
IR	1.3	3.1	2	0.9	1.4	0.1	0	2.8
BW	0.6	0.2	0.4	0.4	0.4	0	0	0.5

C-H：OPE concentrations in homes；C-O：OPE concentrations in offices；C-D：OPE concentrations in student dormitories；C-P：OPE concentrations in private cars.

References

Hartmann, P. C.; Bürgi, D.; Giger, W., Organophosphate flame retardants and plasticizers in indoor air. Chemosphere ***2004,*** *57, (8), 781-787.*

He, C.; Wang, X.; Phong, T.; Baduel, C.; Gallen, C.; Banks, A.; Bainton, P.; English, K.; Mueller, J. F., Organophosphate and brominated flame retardants in Australian indoor environments: Levels, sources, and preliminary assessment of human exposure. Environmental Pollution ***2018,*** *235, 670-679.*

Khairy, M. A.; Lohmann, R., Organophosphate flame retardants in the indoor and outdoor dust and gas-phase of Alexandria, Egypt. Chemosphere ***2019,*** *220, 275-285.*

Kim, U.-J.; Wang, Y.; Li, W.; Kannan, K., Occurrence of and human exposure to organophosphate flame retardants/plasticizers in indoor air and dust from various microenvironments in the United States. Environment International ***2019,*** *125, 342-349.*

Staaf, T.; Ostman, C., Organophosphate triesters in indoor environments. Journal of Environmental Monitoring ***2005,*** *7, (9), 883-887.*

Tokumura, M.; Hatayama, R.; Tatsu, K.; Naito, T.; Takeda, T.; Raknuzzaman, M.; Habibullah-Al-Mamun, M.; Masunaga, S., Organophosphate flame retardants in the indoor air and dust in cars in Japan. Environmental Monitoring and Assessment ***2017,*** *189, (2).*

Wang, Y.; Zhang, Z.; Tan, F.; Rodgers, T. F. M.; Hou, M.; Yang, Y.; Li, X., Ornamental houseplants as potential biosamplers for indoor pollution of organophosphorus flame retardants. Science of the Total Environment ***2021,*** *767.*

Yadav, I. C.; Devi, N. L.; Kumar, A.; Li, J.; Zhang, G., Airborne brominated, chlorinated and organophosphate ester flame retardants inside the buildings of the Indian state of Bihar: Exploration of tag source and human exposure. Ecotoxicology and Environmental Safety ***2020,*** *191.*

Zhou, L.; Hiltscher, M.; Gruber, D.; Puettmann, W., Organophosphate flame retardants (OPFRs) in indoor and outdoor air in the Rhine/Main area, Germany: comparison of concentrations and distribution profiles in different microenvironments. Environmental Science and Pollution Research ***2017,*** *24, (12), 10992-11005.*
